# Environmental and Other Extrinsic Risk Factors Contributing to the Pathogenesis of Cutaneous T Cell Lymphoma (CTCL)

**DOI:** 10.3389/fonc.2019.00300

**Published:** 2019-04-18

**Authors:** Feras M. Ghazawi, Nebras Alghazawi, Michelle Le, Elena Netchiporouk, Steven J. Glassman, Denis Sasseville, Ivan V. Litvinov

**Affiliations:** ^1^Division of Dermatology, University of Ottawa, Ottawa, ON, Canada; ^2^Division of Dermatology, McGill University, Montréal, QC, Canada

**Keywords:** cutaneous T-cell lymphoma, CTCL, epidemiology, incidence, environmental risk factors

## Abstract

The applications of disease cluster investigations in medicine have developed rather rapidly in recent decades. Analyzing the epidemiology of non-random aggregation of patients with a particular disease fostered identification of environmental and external exposures as disease triggers and promoters. Observation of patient clusters and their association with nearby exposures, such as Dr. John Snow's astute mapping analysis in the mid-1800's, which revealed proximity of cholera patients in London to a contaminated water pump infected with *Vibrio cholerae*, have paved the way for the field of epidemiology. This approach enabled the identification of triggers for many human diseases including infections and cancers. Cutaneous T-cell lymphomas (CTCL) represent a group of non-Hodgkin lymphomas that primarily affect the skin. The detailed pathogenesis by which CTCL develops remains largely unknown. Notably, non-random clustering of CTCL patients was reported in several areas worldwide and this rare malignancy was also described to affect multiple members of the same family. These observations indicate that external factors are possibly implicated in promoting CTCL lymphomagenesis. Here, we review the epidemiology of CTCL worldwide and the clinical characteristics of CTCL patients, as revealed by global epidemiological data. Further, we review the known risk factors including sex, age, race as well as environmental, infectious, iatrogenic and other exposures, that are implicated in CTCL lymphomagenesis and discuss conceivable mechanisms by which these factors may trigger this malignancy.

## Cutaneous T-cell Lymphoma

Cutaneous T-cell lymphomas (CTCL) are a class of non-Hodgkin lymphomas. CTCL represents a heterogeneous group of lymphoproliferative disorders that are characterized by the infiltration of activated bystander and malignant CLA^+^ CCR4^+^ T cells into the skin (1, 2). Patients with CTCL, depending on the disease subtype, can present with a spectrum of clinical morphologies including erythematous, hyper- or hypo-pigmented patches with or without atrophy, or present with thickened plaques, that may mimic benign, inflammatory, or autoimmune disorders such as eczema, psoriasis, morphea, pityriasis lichenoides chronica, pityriasis rubra pilaris, drug eruptions, poikiloderma, panniculitis, vitiligo, and pigmented purpuric dermatoses. Thus, CTCL is considered a “great mimicker” in dermatology. In fact, CTCL is often challenging to diagnose, especially in early and erythrodermic stages, and on average it takes ~6 years to establish a definitive diagnosis since its initial presentation (3). As the malignancy progresses in a subset of patients, the disease can spread to lymph nodes and other organs. While mycosis fungoides (MF) and Sézary syndrome (SS) are the most commonly recognized subtypes, other variants of CTCL were documented by the 2016 World Health Organization classification of primary cutaneous lymphomas (4) and include angioimmunoblastic T-cell lymphoma, subcutaneous panniculitis-like T-cell lymphoma, adult T-cell leukemia/lymphoma (ATLL), mature T-cell lymphoma not otherwise specified (NOS), cluster of differentiation 30 positive (CD30^+^) T-cell lymphoproliferative disorders of the skin and extranodal natural killer (NK)/T-cell lymphoma, nasal type (ENKL). Despite the aforementioned variability in clinical presentation, the majority of MF lesions develop on the trunk and lower extremities often asymmetrically and follow a “1930's bathing suit” distribution on skin not commonly exposed to the sun.

## Demographics and Clinical Characteristics of CTCL Patients

While CTCL has been shown to affect individuals of almost all ethnicities and age groups in both sexes, extensive epidemiological studies have consistently reported that CTCL preferentially affects patients of “typical” profiles and in classic body sites. Indeed, the mean age at the time of diagnosis is in the mid- to late-fifties. The mean age of diagnosis was reported in Canada as 59.4 years (5) and the median age of diagnosis in the United States, United Kingdom and Switzerland is in the range of 54–57.5 years, with marked differences between different ethnicities (6–9). In addition, more males are diagnosed with CTCL than females. In fact, the reported incidence-rate-ratio (IRR) of males-to-female patients in the United States during 2001–2005 and 2005–2009 years were 1.7:1 and 1.6:1, respectively (10, 11). The IRR of males-to-females in Canada during 1992–2010 was 1.4:1 (5). There are also important differences in CTCL incidence and prognosis between different ethnic backgrounds. The average age of CTCL diagnosis in African-Americans is significantly lower compared to Caucasians (6–9, 12). In one study that examined the clinical characteristics of 4,496 patients diagnosed with cutaneous lymphoma between 2004 and 2008 in the United States, the mean age at the time of diagnosis for Caucasian, African-American Asian/Pacific Islander, and Native American/other/unknown patients were 59.2, 51.5, 51.3, and 53.8 years, respectively (9). In fact, CTCL was reported to occur at a significantly higher rate in individuals of African-American descent, when compared to individuals of European or Asian origin. In a population-based study in the United States, the age-adjusted incidence rates of CTCL in 8 states from 2001 to 2005 were the highest among African-Americans (10.0/1,000,000 person-years), followed by non-Hispanic whites (8.1), Hispanic whites (5.1), and Asian/Pacific Islander (5.1) (10). Further, in these individuals, the disease was often associated with worse prognosis (9, 12).

In terms of subtype prevalence, MF, SS, CD30^+^ lymphoproliferative disorders and primary cutaneous peripheral T cell lymphomas not otherwise specified (PCTCL-NOS) are the most common and well-recognized variants of CTCL. Indeed, MF is the predominant subtype of CTCL and is corroborated by several studies showing that it accounts for 44–60% of all CTCL cases (1, 8).

## Overall Incidence Patterns for CTCL

The incidence of CTCL worldwide has demonstrated a 2 to 3-fold increase during the last 25–30 years (9, 13), and ranged between ~5 and 11 cases per million individuals per year depending on the CTCL subtypes and the examined year range. In the United States, incidence rates of CTCL were on the rise from 1973 until 1998 and stabilized at 10.2 cases per million individuals per year (11). In Canada, we recently reported that the incidence rate of CTCL increased steadily during the 1990s and then stabilized at ~11 cases per million individuals per year, demonstrating geographical continuity between the two North American countries (5). The incidence rates of CTCL in other parts of the world including Norway, Wales, France, Kuwait, and Japan are summarized in Table 1.

**Table 1:** Reported CTCL incidence rates worldwide (rates are denoted as cases per 1 million individuals per year).

**Country**	**Incidence of CTCL**	**Incidence of CTCL subtypes**	**Study year range**	**Data source**	**References**
USA	2.8–10.5		1973–2009	SEER (6,230 patients from Los Angeles, California; San Jose, California; Alaska and rural Georgia)	(11)
USA		*MF* = 5.5 *SS* = 0.1	2005–2008	SEER program (17 data sets representing ~10% of the US population: Connecticut, Hawaii, Iowa, New Mexico, Utah, Detroit, San Francisco/Oakland, Seattle/Puget Sound and Atlanta)	(14)
USA	6.4		1973–2002	SEER; 13 registries covering ~14% of the US population (Los Angeles and San Jose, California; Alaska; and rural Georgia).	(13)
USA	7.7	*MF* = 4.1 *SS* = 0.1 CD30 + LPD = 1.1 *SPTC* = 0.1 *pcPTL* = 2.2	2001–2005	16 SEER registries representing ~26% of the US population (Connecticut, Hawaii, Iowa, Kentucky, Louisiana, New Jersey, New Mexico, and Utah), greater California, rural Georgia, and 6 metropolitan areas (Atlanta, Detroit, Los Angeles, San Francisco–Oakland, San Jose–Monterrey, and Seattle–Puget Sound)	(10)
USA		MF 1973–1974 = 1.8 1975–1976 = 2.3 1977–1978 = 2.8 1979–1980 = 2.8 1981–1982 = 4.1 1983–1984 = 4.8 1985–1986 = 4.3 1987–1988 = 4.2 1989–1990 = 4.9 1991–1992 = 4.5	1973–92	SEER (specific states not mentioned)	(15)
Canada	11.3		1992–2010	Canadian Cancer Registry and Le Registre Québécois du Cancer	(5)
Norway	1.6 (1980–84) 2.9 (2000–03)	*MF/SS* = 1.5–1.8	1980–2003	Cancer Registry of Norway on non-Hodgkin lymphomas	(16)
France	2.0–5.7	*MF* = 1.3–2.5	1980–2003	Doubs cancer registry (France)	(17)
Kuwait		*MF* = 4.3	1991–2006	National Dermatology Department (193 patients)	(18)
Wales	4.8		2003–2011	All Wales Lymphoma Panel (120 Patients)	(19)
Japan		*MF/SS* = 1.0–1.5	2008–2015	National Cutaneous Lymphoma Registry (391 patients)	(20)

*SEER, Surveillance, Epidemiology, and End Results; MF, mycosis fungoides; SS, Sézary syndrome; CD30^+^ LPD, CD30^+^ T-cell lymphoproliferative disorders of the skin; SPTCL, Subcutaneous panniculitis-like T-cell lymphoma; pcPTL, Primary cutaneous peripheral T-cell lymphoma; AITCL, Angioimmunoblastic T-cell lymphoma; MTCL-NOS, Mature T-cell lymphoma NOS; CD30^+^ALCL, CD30-positive anaplastic large-cell lymphoma; ENKTL-NT, Extranodal NK/T-cell lymphoma, nasal type; ATCLL, Adult T-cell leukemia/lymphoma; EBVANK/TCL, Epstein-Barr virus-associated natural-killer/T-cell lymphoma*.

## Clustering of CTCL Patients

Despite the rarity of this malignancy, clustering of CTCL patients was reported in several regions around the world including the Västernorrland county of Sweden (21) as well as in Pittsburgh, Pennsylvania (22) and in Texas (23) in the United States. Based on the analysis of 1990 patients using two cancer registries (MD Anderson Cancer Center and Texas Cancer Registry), we previously reported geographic clustering of patients in several communities in the state of Texas including in cities of Katy, Beaumont, and Spring, where CTCL incidence rates were 3–20 times higher than the US national average (23, 24). Notably clustering of patients was observed in our recent study in Canada in the areas of heavy industrial presence (e.g., in Winnipeg, MB, Hamilton and Oakville, ON, etc.), while cities with limited industrial presence including Ottawa, Gatineau and Quebec City were relatively spared by CTCL (5). Similarly, Moreau et al. demonstrated CTCL case clustering in certain location of the Pittsburgh metropolitan area (22). Further, familial aggregation of this rare malignancy were reported among Jewish nationals in Israel (25). The non-random distribution of CTCL patients argues that external environmental, occupational, behavioral, and communicable triggers may indeed exist for this malignancy.

## Environmental and External Risk Factors of CTCL

Analysis of the epidemiology and spatial distribution of diseases enabled the identification of occupational, communicable and environmental exposures as initiators/promoters of many malignancies, such as arsenic triggering cutaneous squamous cell carcinomas, benzene exposure contributing to the carcinogenesis of Acute Myelogenous Leukemia, and asbestos accounting for the majority of mesothelioma cases, reviewed in Ghazawi et al. (26, 27).

A number of external triggers/disease promoting factors such as hydrochlorothiazide diuretics, immunosuppression, as well as several putative bacterial and viral agents have been proposed for CTCL (28) and are summarized in Supplementary Table 1. Early evidence shows that air pollution and chemical exposure, including pesticides (e.g., Glyphosate in Roundup®, Monsanto Inc.) and detergents may increase one's risk of developing MF, SS, and other Non-Hodgkin Lymphomas (29, 30). Indeed, as mentioned above, our previous analysis on the distribution of CTCL patient clusters in Texas revealed that many of the patients were residing along the same streets/highways and/or rivers/streams. Similarly, analysis of CTCL distribution by postal codes in Canada identified several regions with elevated CTCL incidence rates that were located in the proximity to heavy industrial factories and major transportation hubs (5, 31). The same trend was observed by Moreau et al. in Pittsburgh, PA area (22). These combined results argue that common exposures may be promoting this cancer in certain communities. Furthermore, our study in Texas demonstrated that two densely populated adjacent zip codes located in a sunny desert climate near El Paso, Texas were completely spared by CTCL (23, 24).

In other population-based studies in the United States, low household income, types of owner-occupied housing units and foreign birth (although, the countries of birth were not detailed in the cited study) were correlated with increased incidence of CTCL (11). Incidence of CTCL has also been correlated with high physician density (correlation coefficient (r) = 0.6; *p* = 0.04) and high family income (*r* = 0.7; *p* = 0.01) (13). In addition, body mass index, tobacco use, personal history of eczema, family history of multiple myeloma, crop, and vegetable farming activities, painting, woodworking and carpentering occupations have all been linked to an increased risk of MF and SS. Alcohol use and sun exposure were also reported as exacerbating and protective lifestyle risk factors for MF, respectively (32). Regarding sun exposure being a protective factor, one plausible hypothesis is centered on low vitamin D levels in CTCL patients. A study by Talpur et al. reported that low vitamin D levels were present in 76.9% of the MF/SS patients, comparable to the levels in other cancer patients (75.2%) (33).

As mentioned earlier, iatrogenic immunosuppression with conventional systemic or newer biologic (i.e., anti TNF-α) therapies increases ones likelihood of developing MF/SS and other lymphomas (28). The use of hydrochlorothiazide was also evaluated in MF and SS patients with hypertension and was found to be a possible trigger of disease in a subset of patients with early MF (34). Although not proving causality, hydrochlorothiazide use has been linked to increased severity in MF and SS cases. The discontinuation of hydrochlorothiazide in these patients has led to the clearing or amelioration of their MF; when re-challenged with this medication, a subset of these patients had a reoccurrence of their MF lesions (34). Other medications that were proposed as possible triggers for MF include antihistamines, antiepileptics, antihypertensives, and serotonin reuptake inhibitors (28).

Familial clustering studies showed an increased incidence of CTCL by analyzing the allele frequency of HLA DQB1^*^03 in first-degree relatives (25). Furthermore, a few cases have reported that organ transplant recipients (albeit these patients are on immunosuppressive drugs) (35) and patients with HIV-related immunodeficiency had an increased risk of developing CTCL (36).

Based on current literature, infections may play more than one role in natural CTCL disease course. Specifically, some infections were proposed to trigger/promote the disease. At the same time, as the malignancy progresses to more advanced stages the host becomes susceptible to an increasing number of infections that ultimately lead to a demise of a patient. Several studies reported a significant incidence of skin infections in CTCL patients with an association between the pathogenic burden and disease severity (37, 38). *Staphylococcus aureus, Mycobacterium leprae, Chlamydophila pneumoniae*, and dermatophytes are among some of the infectious agents implicated as triggers/promoters of CTCL. Moreover, certain viruses investigated for their role in triggering CTCL include *Human T-cell leukemia/lymphotropic virus type 1 (HTLV-1)*, which was consistently associated with Adult T-Cell Lymphoma (39), but not MF/SS (40–46). Further, *HTLV-2* (41, 47), *HIV* (36, 48, 49), *Epstein-Barr virus* (50–61), *Cytomegalovirus* (62, 63), *Human Herpesvirus (HHV)-6, HHV-7* (57, 64–66), *HHV-8* (67) and even *Polyomaviruses* including *Merkel cell polyomavirus (MCV)* (68–70) were also proposed to play an important role in disease pathogenesis. However, some of these studies have yielded conflicting results, as highlighted by Mirvish et al. (71), and ultimately failed to report a clear explanation for CTCL pathogenesis (71).

## How Could External Factors Promote or Trigger CTCL?

While the precise triggers are not yet identified/confirmed, and the mechanism of lymphomagenesis remains enigmatic, several studies have investigated a number of different hypotheses (72). Chromosomal instability as well as dysregulated expression of many genes such as cancer testis and meiomitosis genes, Suppressor of cytokine signaling 3 (SOCS3), B-Raf proto-oncogene, serine/threonine kinase (BRAF), Interleukin-2 receptor common gamma chain, Thymocyte selection-associated high-mobility group box (TOX), among others [reviewed in (72, 73)] were reported in CTCL patients. Aberrant expression of SOCS3, a regulator of the Jak-3/STAT disrupts the normal expression of several cytokines including IL-5, IL-10, IL-17A, and IL-17F and tumor suppressor microRNAs such as miR-22 further highlighting the important role of the cytokine milieu in disease pathogenesis (74). As disease progresses, an important switch from a Th1 to Th2 profile immune response is observed in patients with subsequent eosinophilia and superinfections with *S. aureus* (75, 76). On the other hand, a recent study by Fanok et al. demonstrated that T-cell receptor engagement is critical for malignant transformation of T lymphocytes in the setting of presumed bacterial trigger (77). *S. aureus*, being a common pathogen residing on the skin, is thought to promote malignant inflammation. Lack of Th1 immune response is a driving factor for the growth of *S. aureus* on the CTCL skin. Following this event, a Th2 immune-mediated response is precipitated by the downregulation of STAT4 and upregulation of STAT5 and STAT3 by oncomiRs (miR-155) making CTCL patients more susceptible to *S. aureus* colonization and prolonged antigenic stimulation (75, 76, 78, 79).

Further, a recent study described the mechanism by which *S. aureus* activates oncogenic STAT3 signaling in malignant T cells (80). Staphylococcal enterotoxin A (SEA) was shown to impact malignant T cells in an indirect mechanism by promoting infiltrating bystander non-malignant T cells to produce IL-2 and other regulatory cytokines, thus upregulating JAK3/STAT3 signaling and leading to proliferation of malignant T cells in the skin (28, 80). Finally, Staphylococcal toxins have been implicated in the activation of malignant T cells in SS patients by acting as superantigens and binding to a TCR-v β chain, leading to T cell activation and proliferation (81–83). Collectively, it is likely that patients' genetic profiles and skewed cytokine milieu in response to select infectious agents may predispose individuals to develop MF/SS.

Also, central to CTCL pathogenesis remains chronic and persistent antigen stimulation of skin-homing CD4^+^ memory T-cells by external factors. The resultant chronic inflammation drives the immune system toward the proliferation and expansion of specific T cell malignant clone(s) (84). This was reinforced by a collection of different observations. For instance, it was reported that one patient, who had implanted gravel in the hip from a traumatic injury developed a chronic plaque, which histologically appeared as parapsoriasis and progressed in 15 years to MF (85). The fact that, after about 10 years on average, some patients developed MF at the sites previously inoculated with foreign substances also supports the notion that CTCL may develop after chronic antigen stimulation (86). Consistent with these observations, it is well-established that implant associated-Anaplastic Lage Cell Lymphoma (ALCL), an indolent disease similar to primary cutaneous ALCL, can arise as a result of breast implants, tibial implants, dental implants, chest injection ports, gluteal implants, shoulder repairs, and a gastric band placement (87). It is now standard procedure to include a discussion of ALCL risk before any breast implant procedures and inform patients of a possible onset of ALCL within a median time frame of 8 to 10 years, albeit this disease affects 1 in 2,000 to 1 in 70,000 implant recipients (87). Notably, textured prostheses have higher risk of ALCL then smooth implants (87).

The reviewed implicated factors in Supplementary Table 1 clearly indicate that more than one antigen may stimulate CTCL. Additional evidence of this comes from studies that established a widespread repertoire of the stimulated CTCL clones (or single-cell heterogeneity in Sézary Syndrome) between different patients (88). Therefore, particular antigen engagement, coupled with the combination of aberrant cytokine milieu and chronicity of antigenic-stimulation, may collectively play critical roles in the development of cutaneous lymphoma.

## Conclusions

Epidemiological studies have repeatedly helped identify definitive triggers for several diseases. As highlighted in this perspective report, previous studies strongly argue for the interplay between intrinsic factors and putative preventable extrinsic triggers/promoters for CTCL. Given the evidence of geographical regional clustering of CTCL patients, CTCL occurrence in unrelated family members and recent evidence implicating *S. aureus* in the pathogenesis/progression of CTCL, more research is needed to decipher the precise mechanism by which specific environmental exposures may be driving the pathogenesis of this malignancy. Hopefully, such knowledge of potential triggers and perpetuating factors for this cancer would enable us at some point to significantly decrease CTCL incidence. Therefore, it is important to take into consideration the effects of hydrochlorothiazide diuretics and immunosuppressive medications in patients with definite or suspected diagnosis of CTCL (e.g., recalcitrant eczematous patches/plaques that appear in a bathing-suit distribution) (89). Patients living near heavy industrial factories may consider an air filtration system, if feasible, to decrease their risk of developing a malignancy. Minimizing daily exposure to pesticides/herbicides containing chemicals that are listed as probable or possible (Groups 1-2B) carcinogens on the International Agency for Research on Cancer (IARC) database, such as glyphosate, is also important. Any traumatically implanted in the skin or subcutaneous tissue foreign objects (especially textured objects) should be surgically removed. Furthermore, given our further understanding of *S. aureus* in the pathogenesis of CTCL progression, clinicians may consider decolonizing patients using various techniques already used for patients with atopic dermatitis such as bleach baths as well as topical and systemic antibiotics. Similarly, patients with detectable dermatophytes may similarly benefit from anti-fungal treatments to avoid developing CTCL.

## Author Contributions

FG, NA, ML, EN, SG, DS, and IL have contributed by participating in reviewing the literature on the topic, preparing the tables summarizing existing evidence, writing and critically reviewing the manuscript.

### Conflict of Interest Statement

The authors declare that the research was conducted in the absence of any commercial or financial relationships that could be construed as a potential conflict of interest.
