# Supplementary material for: Environmental and Other Extrinsic Risk Factors Contributing to the Pathogenesis of Cutaneous T Cell Lymphoma (CTCL)
Source: Front Oncol. 2019 Apr 18;9:300. doi: 10.3389/fonc.2019.00300 (PMC6499168; doi:10.3389/fonc.2019.00300)
Supplement: Supplementary file 1 [file Table_1.DOCX]

**Supplementary Table 1.** Summary of studies documenting associations between various extrinsic and extrinsic risk factors for CTCL.

| **Country** | **Implicated risk factors of CTCL** | **CTCL subtype** | **Relative risk/ Study Results** | **References** |
| --- | --- | --- | --- | --- |
| USA | Pollutants air exposure and chemicals | MF, SS | History of multiple exposures to chemicals was reported (in 91% of patients) and drugs (86%). Air pollutants (39%), pesticides (36%), solvents and vapors (30%), and detergents and disinfectants (14%) analgesics (20%), tranquilizers (18%) and thiazides (14%).  Patient population: 44 patients with cutaneous T-cell lymphoma from the National Cancer Institute therapeutic trial. | (Fischmann et al., 1979) |
| USA, Canada | Contaminated water supply, air pollution, or industrial exposure (oil refineries), inciting infectious, radioactive, or chemical agent exposure | MF, SS | Hypothesized triggers due to geographical proximity of industrial presence such as oil refineries. | (Litvinov et al., 2015a;Litvinov et al., 2015b;Phan et al., 2016;Ghazawi et al., 2017) |
| Iran | Sulphur mustard (SM) | MF | In comparison to MF incidence rate in Iranian general population (0.39/100,000 person-years), the incidence rate of patients with exposure to SM is 0.80/100,000 person-years. | (Emadi et al., 2017) |
| South Korea | Agent Orange | MF | There was a total of 12 MF patients with a history of Agent Orange exposure. The patients differed from the control group with respect to age, sex, symptoms and morphology of lesions including lichenification. | (Jang et al., 2013) |
| USA | Hydrochlorothiazide (HCTZ) | MF, SS | 65% of CTCL patients with hypertension taking HCTZ, either started the drug prior to developing MF or their MF increased in severity once they started HCTZ  Patient population: 1443 confirmed MF and SS patients. | (Fischmann et al., 1979;Jahan-Tigh et al., 2013) |
| USA | History of cigarette smoking ≥ 40years | MF, SS | OR = 1.55, 95% CI = 1.04 to 2.31 | (Phan et al., 2016) |
| USA | Sun exposure as a protective factor | Anaplastic large cell lymphoma | OR = 0.48, 95% CI = 0.26 to 0.88 | (Phan et al., 2016) |
| Spain | Alcohol use | MF | OR=3.02, 95% confidence interval (CI), 1.34 to 6.79 | (Morales Suarez-Varela et al., 2001) |
| USA | Foreign born nationality | CTCL | r = 0.8, P = .001 | (Korgavkar et al., 2013) |
| USA | Body mass index ≥ 30 kg/m^2^ | MF, SS | OR = 1.57, 95% CI = 1.03 to 2.40 | (Aschebrook-Kilfoy et al., 2014) |
| USA | Body mass index ≥ 30 kg/m^2^ | MF, SS | OR = 1.57, 95% CI = 1.03 to 2.40 | (Phan et al., 2016) |
| USA, | High population/ dermatologists’ density | CTCL, MF | Geographic cluster (p<.001) in zip code 15213 in Pittsburgh. | (Weinstock and Gardstein, 1999;Moreau et al., 2014) |
| USA | Moderate leisure time physical activity | MF, SS | OR = 0.46, 95% CI = 0.22 to 0.97 | (Phan et al., 2016) |
| USA | History of celiac disease | Anaplastic large cell lymphoma | OR = 16.59, 95% CI = 3.27 to 84.31 | (Phan et al., 2016) |
| USA | History of eczema | MF, SS | OR = 8.49, 95% CI = 3.31 to 21.80 | (Phan et al., 2016) |
| USA | History of eczema | MF, SS | OR = 2.38, 95% CI = 1.73 to 3.29 | (Aschebrook-Kilfoy et al., 2014) |
| USA | History of eczema | Anaplastic large cell lymphoma | OR = 1.83, 95% CI = 0.75 to 4.42 | (Phan et al., 2016) |
| USA | History of psoriasis | Anaplastic large cell lymphoma | OR = 3.19, 95% CI = 1.50 to 6.80 | (Phan et al., 2016) |
| USA | Family history of atopy | MF | RR=4.50 95% CI=0.89 to 12.95 | (Tuyp et al., 1987) |
| USA | Family history of multiple myeloma | MF, SS | OR = 8.49, 95% CI = 3.31 to 21.80 | (Aschebrook-Kilfoy et al., 2014;Phan et al., 2016) |
| USA | Family history of hematologic malignancies | Angioimmunoblastic T-cell lymphoma | OR = 2.55, 95% CI = 1.10 to 5.89 | (Phan et al., 2016) |
| Israel | Common viral/ environmental exposure in cases of familial clustering and in married couples | MF | The frequency of HLA DQB1∗03 Allele was significantly greater in MF patients compared to the control group (66.7% vs 33%, respectively; P = .027)  Study population= 300 patients with MF included first-degree relatives. | (Dupin et al., 1995;Hodak et al., 2005;Schmidt et al., 2006;Lozano and Duvic, 2007) |
| USA | Bachelor’s or graduate degree | CTCL | r=0.6, P = .053 | (Korgavkar et al., 2013) |
| USA | High percentage of adults with a bachelor's or graduate degree | CTCL | r = 0.6; P = .02 | (Criscione and Weinstock, 2007) |
| USA | High median value of owner-occupied housing units | CTCL | r = 0.6; P = .02 | (Criscione and Weinstock, 2007) |
| USA | Owner-occupied housing units | CTCL | r = 0.8, P < .001 | (Korgavkar et al., 2013) |
| USA | Household income | CTCL | r = 0.7, P = .01  Patient population: 9 original registries (1973-2009) and the 4 additional registries (1992-2009) of SEER program of the NCI | (Korgavkar et al., 2013) |
| USA | High median family income | CTCL | r = 0.7; P = .01 | (Criscione and Weinstock, 2007) |
| USA | High physician density | CTCL | r = 0.6; P = .04  Patient population: A total of 4783 cases of CTCL between 1973 to 2002 in 13 SEER registries. | (Criscione and Weinstock, 2007) |
| USA | High density of medical specialists | CTCL | r = 0.7; P = .02 | (Criscione and Weinstock, 2007) |
| USA | African-American ethnicity/race | Angioimmunoblastic T-cell lymphoma | OR = 0.48, 95% CI = 0.38 to 0.61 | (Phan et al., 2016) |
| USA | Asian/Pacific Islander ethnicity/race | Angioimmunoblastic T-cell lymphoma | OR = 1.22, 95% CI = 0.99 to 1.50 | (Phan et al., 2016) |
| USA | Hispanic ethnicity/race | Angioimmunoblastic T-cell lymphoma | OR = 1.02, 95% CI = 0.83 to 1.25 | (Phan et al., 2016) |
| USA | American Indian/Alaskan Native ethnicity/race | Angioimmunoblastic T-cell lymphoma | OR = NE, 95% CI = 0.10 to 1.22 | (Phan et al., 2016) |
| USA | African-American ethnicity/race | Adult T-cell leukemia/lymphoma | OR = 2.58, 95% CI = 2.01 to 3.31 | (Phan et al., 2016) |
| USA | Asian/Pacific Islander ethnicity/race | Adult T-cell leukemia/lymphoma | OR = 1.92, 95% CI = 1.31 to 2.78 | (Phan et al., 2016) |
| USA | Hispanic ethnicity/race | Adult T-cell leukemia/lymphoma | OR = 1.23, 95% CI = 0.83 to 1.82 | (Phan et al., 2016) |
| USA | African American ethnicity/race | Extranodal natural kill/T-cell lymphoma | OR = 0.42, 95% CI = 0.30 to 0.61 | (Phan et al., 2016) |
| USA | Asian/Pacific Islander ethnicity/race | Extranodal natural kill/T-cell lymphoma | OR = 3.29, 95% CI = 2.59 to 4.19 | (Phan et al., 2016) |
| USA | Hispanic ethnicity/race | Extranodal natural kill/T-cell lymphoma | OR = 3.55, 95% CI = 2.90 to 4.35 | (Phan et al., 2016) |
| USA | American Indian/Alaskan Native ethnicity/race | Extranodal natural kill/T-cell lymphoma | OR = 3.92, 95% CI = 1.98 to 7.76 | (Phan et al., 2016) |
| USA | African-American ethnicity/race | Anaplastic large cell lymphoma | OR = 0.66, 95% CI = 0.56 to 0.77 | (Phan et al., 2016) |
| USA | Asian/Pacific Islander ethnicity/race | Anaplastic large cell lymphoma | OR = 0.62, 95% CI = 0.49 to 0.77 | (Phan et al., 2016) |
| USA | Hispanic ethnicity/race | Anaplastic large cell lymphoma | OR = 1.03, 95% CI = 0.87 to 1.22 | (Phan et al., 2016) |
| USA | American Indian/Alaskan Native ethnicity/race | Anaplastic large cell lymphoma | OR = 1.51, 95% CI = 0.83 to 2.76 | (Phan et al., 2016) |
| USA | Woodworkers | MF, SS | OR = 2.20, 95% CI = 1.18 to 4.08 | (Aschebrook-Kilfoy et al., 2014) |
| USA | General carpenters | MF, SS | OR = 4.07, 95% CI = 1.54 to 10.75 | (Aschebrook-Kilfoy et al., 2014) |
| USA | Electrical fitters | Angioimmunoblastic T-cell lymphoma | OR = 5.45, 95% CI = 1.20 to 24.7 | (Phan et al., 2016) |
| USA | Crop and vegetable farmers | MF, SS | OR = 2.37, 95% CI = 1.14 to 4.92 | (Aschebrook-Kilfoy et al., 2014) |
| USA | Painters | MF, SS | OR = 3.71, 95% CI = 1.94 to 7.07 | (Aschebrook-Kilfoy et al., 2014) |
| USA | Textile workers | Anaplastic large cell lymphoma | OR = 2.60, 95% CI = 1.21 to 5.58 | (Phan et al., 2016) |
| USA | Electrical fitters | Anaplastic large cell lymphoma | OR = 4.08, 95% CI = 1.36 to 12.2 | (Phan et al., 2016) |
| USA | Occupation as crop or vegetable farmer | MF, SS | OR = 2.37, 95% CI = 1.14 to 4.92 | (Phan et al., 2016) |
| USA | Occupation as painter | MF, SS | OR = 3.71, 95% CI = 1.94 to 7.07 | (Phan et al., 2016) |
| USA | Occupation as woodworker | MF, SS | OR = 2.20, 95% CI = 1.18 to 4.08 | (Phan et al., 2016) |
| USA | Occupation as general carpenter | MF, SS | OR = 4.07, 95% CI = 1.54 to 10.75 | (Phan et al., 2016) |
| USA | Organ transplant recipients/iatrogenic immunosuppression | CTCL | Incidence of CTCL seems increased among transplant recipients receiving immunosuppressive medications. | (Pomerantz et al., 2010) |
| USA | Genetic susceptibility (Human leukocyte class II antigen DRB1*11) | MF, SS | HLA-DR5- odds ratio OR = 3.62, 1.9 < OR < 10 in MF and OR = 3, 0.9 < OR < 9.3 in SS, HLA-DQB1*03 alleles - OR = 2.15, 1 < OR < 4.5 in MF and OR = 4.7, 1.4 < OR < 5 in SS  Patient population: MF (n = 47) and SS (n = 23). | (Safai et al., 1983;Detmar et al., 1991;Jackow et al., 1996;Hodak et al., 2005) |
| USA, Italy | Multiple infectious agents | MF | 21/83 cases had concurrent presence of 2/3 pathogens compared to 1/83 in control group (Bonin et al., 2010).  Superantigen activity is implied due to the dominance of a single V beta family in the polyclonally expanded dermal T-cell populations in the CTCL lesions (Linnemann et al., 2004). | (Tan et al., 1974;Linnemann et al., 2004;Bonin et al., 2010) |
| USA | Human T-cell leukemia/lymphotropic virus type 1 (HTLV-I) | MF | 4/9 (44%) Positive samples for HTLV-I DNA (Zucker-Franklin et al., 1992).  HTLV pol and/or tax proviral sequences in 46/50 (92%) (Pancake et al., 1995).  11/12 patients detected HTLV-I proviral sequences in their circulating lymphocytes via PCR (Khan et al., 1996).  12/28 patients positive for HTLV-1 antibodies tested via Western blot (Shohat et al., 1999). | (Hall et al., 1991;Zucker-Franklin et al., 1992;Pancake et al., 1995;Khan et al., 1996;Pancake et al., 1996;Shohat et al., 1999) |
| USA | HTLV-II | MF | 1/9 (9.8%) Samples positive for HTLV-II DNA (Zucker-Franklin et al., 1992).  46/50 (92%) of patients had HTLV pol and/or tax proviral sequences (Pancake et al., 1995), | (Zucker-Franklin et al., 1992;Poiesz et al., 2000) |
| USA | Human-immunodeficiency-virus (HIV) infection | CTCL, MF | RR of T-cell lymphoma estimated was 15.0 (95% confidence interval: 10.0-21.7). The risks were increased for all subtypes, including mycosis fungoides, peripheral lymphomas, cutaneous lymphomas, and adult T-cell leukemia/lymphoma (ATLL) (Biggar et al., 2001). | (Bachelez et al., 1996;Biggar et al., 2001;Wilkins et al., 2006) |
| Germany, Turkey | Human Herpesvirus (HHV) 8 | MF | 7/10 (70%) MF samples positive for KSHV/HHV-8 DNA (Kreuter et al., 2008). | (Erkek et al., 2002;Kreuter et al., 2008) |
| USA, France, Spain, Pakistan, Italy, Korea, Switzerland | Epstein-Barr virus | MF, SS | 7/29 (24%) MF samples positive for EBV DNA (Nagore et al., 2000).  2/4 (50%) Pleomorphic small/medium CTCL samples positive for EBV DNA (Nagore et al., 2000).  13 of 21 CTCL patients had detectable antibodies to Epstein-Barr Nuclear Antigens (EBNA), and only 5/20 control psoriatic patients were CIF positive (Lee et al., 1990).  37 patients had anti–CVA (Anti–EBV antibodies) ≥ 1200 vs. 19 controls (P<0.01) (Jumbou et al., 1998).  EBV-DNA-positive in 8/30 (27%) SS, 7/71 (10%) MF, and 2/18 (11%) (Novelli et al., 2009). | (Lee et al., 1990;Borisch et al., 1992;Dreno et al., 1994;Cho et al., 1996;Mouly et al., 1996;Jumbou et al., 1997;Jumbou et al., 1998;Nagore et al., 2000;Tournadre et al., 2001;Noorali et al., 2002;Novelli et al., 2009;Park et al., 2010) |
| USA | Cytomegalovirus | MF, SS | Of 116 MF/SS CTCL patients, 113 (97.4%) had positive CMV-IgG serologies, MF/SS patients as a group had a significantly higher rate of CMV seropositivity compared with healthy control subjects (Herne et al., 2003).  CMV seroprevalence was 66.67% in the MF group (P = 0.009), 42.86% in the SS group (P = 0.9) (Ballanger et al., 2009). | (Herne et al., 2003;Ballanger et al., 2009) |
| USA, Italy | Human Herpesvirus (HHV) HHV-6 and HHV-7 | MF, SS, CD30 large-cell CTCL | 6/33 (18%) PCR Samples positive for HHV-7 DNA (Nagore et al., 2000).  21/148 (14.2%) Samples positive for HHV-7 DNA (Ponti et al., 2008). | (Brice et al., 1993;Nagore et al., 2000;Erkek et al., 2002;Ponti et al., 2008) |
| Germany | HHV-8 | MF | 7/10 (70%) MF samples positive for KSHV/HHV-8 DNA(Kreuter et al., 2008) | (Kreuter et al., 2008) |
| USA, Germany | Polyomaviruses including Merkel cell polyomavirus (MCV) | CTCL | 4 of 23 (17.4%) had MCPyV DNA detection (Andres et al., 2010).  MCV LT expression of protein localized to nuclei of tumor cells from MCC having PCR quantified MCV genome with an average of 5.2 (range 0.8–14.3) T antigen DNA copies per cell (Shuda et al., 2009). | (Shuda et al., 2009;Andres et al., 2010;Kreuter et al., 2011) |
| Italy | Parvoviridae DNA | CTCL | Parvovirus DNA was detected in CTCL patients (34%; n = 76)  Parvovirus DNA detected by PCR in 4/17 CTCL cases and 0/31 controls tested (p=0.022). | (Sidoti et al., 2009) |
| Japan | Staphylococcus aureus | SS | *S. aureus* skin colonization influences the disease activity of CTCL, possibly by activation of Sézary cells by bacterial superantigenic exoproteins (Sidoti et al, 2009).  Superantigen reaction occurs in CTCL cells which implies the possibility of the involvement of bacterial toxins in the pathogenesis of CTCL ([Tokura, Yagi et al. 1995](#_ENREF_76)). | (Tokura et al., 1992;Tokura et al., 1995;Sidoti et al., 2009) |
| USA | Chlamydophila pneumoniae | MF, SS | *C. pneumoniae*, can provide an antigen that stimulates proliferation of Sezary T cells ([Abrams, Balin et al. 2001](#_ENREF_2)). | (Abrams et al., 1999;Abrams et al., 2001) |

Abbreviations: OR odd ratio, RR relative risk, r correlation coefficient, CI confidence intervals, MF Mycosis Fungoides, SS Sézary Syndrome.

**References:**

Abrams, J.T., Balin, B.J., and Vonderheid, E.C. (2001). Association between Sezary T cell-activating factor, Chlamydia pneumoniae, and cutaneous T cell lymphoma. *Ann N Y Acad Sci* 941**,** 69-85.

Abrams, J.T., Vonderheid, E.C., Kolbe, S., Appelt, D.M., Arking, E.J., and Balin, B.J. (1999). Sezary T-cell activating factor is a Chlamydia pneumoniae-associated protein. *Clin Diagn Lab Immunol* 6**,** 895-905.

Andres, C., Puchta, U., Sander, C.A., Ruzicka, T., and Flaig, M.J. (2010). Prevalence of Merkel cell polyomavirus DNA in cutaneous lymphomas, pseudolymphomas, and inflammatory skin diseases. *Am J Dermatopathol* 32**,** 593-598.

Aschebrook-Kilfoy, B., Cocco, P., La Vecchia, C., Chang, E.T., Vajdic, C.M., Kadin, M.E., Spinelli, J.J., Morton, L.M., Kane, E.V., Sampson, J.N., Kasten, C., Feldman, A.L., Wang, S.S., and Zhang, Y. (2014). Medical history, lifestyle, family history, and occupational risk factors for mycosis fungoides and Sezary syndrome: the InterLymph Non-Hodgkin Lymphoma Subtypes Project. *J Natl Cancer Inst Monogr* 2014**,** 98-105.

Bachelez, H., Hadida, F., and Gorochov, G. (1996). Massive infiltration of the skin by HIV-specific cytotoxic CD8+ T cells. *N Engl J Med* 335**,** 61-62.

Ballanger, F., Bressollette, C., Volteau, C., Planche, L., and Dreno, B. (2009). Cytomegalovirus: its potential role in the development of cutaneous T-cell lymphoma. *Exp Dermatol* 18**,** 574-576.

Biggar, R.J., Engels, E.A., Frisch, M., and Goedert, J.J. (2001). Risk of T-cell lymphomas in persons with AIDS. *J Acquir Immune Defic Syndr* 26**,** 371-376.

Bonin, S., Tothova, S.M., Barbazza, R., Brunetti, D., Stanta, G., and Trevisan, G. (2010). Evidence of multiple infectious agents in mycosis fungoides lesions. *Exp Mol Pathol* 89**,** 46-50.

Borisch, B., Boni, J., Burki, K., and Laissue, J.A. (1992). Recurrent cutaneous anaplastic large cell (CD30+) lymphoma associated with Epstein-Barr virus. A case report with 9-year follow-up. *Am J Surg Pathol* 16**,** 796-801.

Brice, S.L., Jester, J.D., Friednash, M., Golitz, L.E., Leahy, M.A., Stockert, S.S., and Weston, W.L. (1993). Examination of cutaneous T-cell lymphoma for human herpesviruses by using the polymerase chain reaction. *J Cutan Pathol* 20**,** 304-307.

Cho, K.H., Kim, C.W., Lee, D.Y., Sohn, S.J., Kim, D.W., and Chung, J.H. (1996). An Epstein-Barr virus-associated lymphoproliferative lesion of the skin presenting as recurrent necrotic papulovesicles of the face. *Br J Dermatol* 134**,** 791-796.

Criscione, V.D., and Weinstock, M.A. (2007). Incidence of cutaneous T-cell lymphoma in the United States, 1973-2002. *Arch Dermatol* 143**,** 854-859.

Detmar, M., Pauli, G., Anagnostopoulos, I., Wunderlich, U., Herbst, H., Garbe, C., Stein, H., and Orfanos, C.E. (1991). A case of classical mycosis fungoides associated with human T-cell lymphotropic virus type I. *Br J Dermatol* 124**,** 198-202.

Dreno, B., Celerier, P., Fleischmann, M., Bureau, B., and Litoux, P. (1994). Presence of Epstein-Barr virus in cutaneous lesions of mycosis fungoides and Sezary syndrome. *Acta Derm Venereol* 74**,** 355-357.

Dupin, M., Darie, H., Jumbou, O., Veran, Y., Gros, P., Dreno, B., Dormont, D., and Millet, P. (1995). [Conjugal mycosis fungoides]. *Ann Dermatol Venereol* 122**,** 595-598.

Emadi, S.N., Shiri, M., Shiri, Z., Emadi, S.E., Mortazavi, H., Nikoo, A., and Akhavan-Moghaddam, J. (2017). Mycosis fungoides two decades after exposure to sulphur mustard: a follow-up of 1100 victims. *J Eur Acad Dermatol Venereol* 31**,** 432-437.

Erkek, E., Senturk, N., Dincer, I., Olut, A.I., Kocagoz, T., Bukulmez, G., and Sahin, S. (2002). Identification of herpes simplex virus DNA and lack of human herpesvirus-8 DNA in mycosis fungoides. *Acta Derm Venereol* 82**,** 214-216.

Fischmann, A.B., Bunn, P.A., Jr., Guccion, J.G., Matthews, M.J., and Minna, J.D. (1979). Exposure to chemicals, physical agents, and biologic agents in mycosis fungoides and the Sezary syndrome. *Cancer Treat Rep* 63**,** 591-596.

Ghazawi, F.M., Netchiporouk, E., Rahme, E., Tsang, M., Moreau, L., Glassman, S., Provost, N., Gilbert, M., Jean, S.E., Pehr, K., Sasseville, D., and Litvinov, I.V. (2017). Comprehensive analysis of cutaneous T-cell lymphoma (CTCL) incidence and mortality in Canada reveals changing trends and geographic clustering for this malignancy. *Cancer* 123**,** 3550-3567.

Hall, W.W., Liu, C.R., Schneewind, O., Takahashi, H., Kaplan, M.H., Roupe, G., and Vahlne, A. (1991). Deleted HTLV-I provirus in blood and cutaneous lesions of patients with mycosis fungoides. *Science* 253**,** 317-320.

Herne, K.L., Talpur, R., Breuer-Mcham, J., Champlin, R., and Duvic, M. (2003). Cytomegalovirus seropositivity is significantly associated with mycosis fungoides and Sezary syndrome. *Blood* 101**,** 2132-2136.

Hodak, E., Klein, T., Gabay, B., Ben-Amitai, D., Bergman, R., Gdalevich, M., Feinmesser, M., Maron, L., and David, M. (2005). Familial mycosis fungoides: report of 6 kindreds and a study of the HLA system. *J Am Acad Dermatol* 52**,** 393-402.

Jackow, C.M., Mcham, J.B., Friss, A., Alvear, J., Reveille, J.R., and Duvic, M. (1996). HLA-DR5 and DQB1*03 class II alleles are associated with cutaneous T-cell lymphoma. *J Invest Dermatol* 107**,** 373-376.

Jahan-Tigh, R.R., Huen, A.O., Lee, G.L., Pozadzides, J.V., Liu, P., and Duvic, M. (2013). Hydrochlorothiazide and cutaneous T cell lymphoma: prospective analysis and case series. *Cancer* 119**,** 825-831.

Jang, M.S., Jang, J.G., Han, S.H., Park, J.B., Kang, D.Y., Kim, S.T., and Suh, K.S. (2013). Clinicopathological features of mycosis fungoides in patients exposed to Agent Orange during the Vietnam War. *J Dermatol* 40**,** 606-612.

Jumbou, O., Huet, S., Bureau, B., Litoux, P., and Dreno, B. (1998). [Epstein-Barr virus research by in situ hybridization in 65 cutaneous T cell epidermotropic lymphomas]. *Ann Dermatol Venereol* 125**,** 90-93.

Jumbou, O., Mollat, C., N'guyen, J.M., Billaudel, S., Litoux, P., and Dreno, B. (1997). Increased anti-Epstein-Barr virus antibodies in epidermotropic cutaneous T-cell lymphoma: a study of 64 patients. *Br J Dermatol* 136**,** 212-216.

Khan, Z.M., Sebenik, M., and Zucker-Franklin, D. (1996). Localization of human T-cell lymphotropic virus-1 tax proviral sequences in skin biopsies of patients with mycosis fungoides by in situ polymerase chain reaction. *J Invest Dermatol* 106**,** 667-672.

Korgavkar, K., Xiong, M., and Weinstock, M. (2013). Changing incidence trends of cutaneous T-cell lymphoma. *JAMA Dermatol* 149**,** 1295-1299.

Kreuter, A., Bischoff, S., Skrygan, M., Wieland, U., Brockmeyer, N.H., Stucker, M., Altmeyer, P., and Gambichler, T. (2008). High association of human herpesvirus 8 in large-plaque parapsoriasis and mycosis fungoides. *Arch Dermatol* 144**,** 1011-1016.

Kreuter, A., Silling, S., Dewan, M., Stucker, M., and Wieland, U. (2011). Evaluation of 4 recently discovered human polyomaviruses in primary cutaneous B-cell and T-cell lymphoma. *Arch Dermatol* 147**,** 1449-1451.

Lee, P.Y., Charley, M., Tharp, M., Jegasothy, B.V., and Deng, J.S. (1990). Possible role of Epstein-Barr virus infection in cutaneous T-cell lymphomas. *J Invest Dermatol* 95**,** 309-312.

Linnemann, T., Gellrich, S., Lukowsky, A., Mielke, A., Audring, H., Sterry, W., and Walden, P. (2004). Polyclonal expansion of T cells with the TCR V beta type of the tumour cell in lesions of cutaneous T-cell lymphoma: evidence for possible superantigen involvement. *Br J Dermatol* 150**,** 1013-1017.

Litvinov, I.V., Tetzlaff, M.T., Rahme, E., Habel, Y., Risser, D.R., Gangar, P., Jennings, M.A., Pehr, K., Prieto, V.G., Sasseville, D., and Duvic, M. (2015a). Identification of geographic clustering and regions spared by cutaneous T-cell lymphoma in Texas using 2 distinct cancer registries. *Cancer* 121**,** 1993-2003.

Litvinov, I.V., Tetzlaff, M.T., Rahme, E., Jennings, M.A., Risser, D.R., Gangar, P., Netchiporouk, E., Moreau, L., Prieto, V.G., Sasseville, D., and Duvic, M. (2015b). Demographic patterns of cutaneous T-cell lymphoma incidence in Texas based on two different cancer registries. *Cancer Med* 4**,** 1440-1447.

Lozano, A., and Duvic, M. (2007). Cutaneous T-cell lymphoma in non-blood-related family members: report of an additional case. *J Am Acad Dermatol* 56**,** 521.

Morales Suarez-Varela, M.M., Olsen, J., Kaerlev, L., Guenel, P., Arveux, P., Wingren, G., Hardell, L., Ahrens, W., Stang, A., Llopis-Gonzalez, A., Merletti, F., Guillen-Grima, F., and Johansen, P. (2001). Are alcohol intake and smoking associated with mycosis fungoides? A European multicentre case-control study. *Eur J Cancer* 37**,** 392-397.

Moreau, J.F., Buchanich, J.M., Geskin, J.Z., Akilov, O.E., and Geskin, L.J. (2014). Non-random geographic distribution of patients with cutaneous T-cell lymphoma in the Greater Pittsburgh Area. *Dermatol Online J* 20.

Mouly, F., Baccard, M., Rybojad, M., Lebbe, C., Morinet, F., and Morel, P. (1996). [Aggressive cutaneous T-cell lymphoma associated with the presence of Epstein-Barr virus. 2 cases]. *Ann Dermatol Venereol* 123**,** 574-576.

Nagore, E., Ledesma, E., Collado, C., Oliver, V., Perez-Perez, A., and Aliaga, A. (2000). Detection of Epstein-Barr virus and human herpesvirus 7 and 8 genomes in primary cutaneous T- and B-cell lymphomas. *Br J Dermatol* 143**,** 320-323.

Noorali, S., Yaqoob, N., Nasir, M.I., Moatter, T., and Pervez, S. (2002). Prevalence of mycosis fungoides and its association with EBV and HTLV-1 in Pakistanian patients. *Pathol Oncol Res* 8**,** 194-199.

Novelli, M., Merlino, C., Ponti, R., Bergallo, M., Quaglino, P., Cambieri, I., Comessatti, A., Sidoti, F., Costa, C., Corino, D., Cavallo, R., Ponzi, A.N., Fierro, M.T., and Bernengo, M.G. (2009). Epstein-Barr virus in cutaneous T-cell lymphomas: evaluation of the viral presence and significance in skin and peripheral blood. *J Invest Dermatol* 129**,** 1556-1561.

Pancake, B.A., Wassef, E.H., and Zucker-Franklin, D. (1996). Demonstration of antibodies to human T-cell lymphotropic virus-I tax in patients with the cutaneous T-cell lymphoma, mycosis fungoides, who are seronegative for antibodies to the structural proteins of the virus. *Blood* 88**,** 3004-3009.

Pancake, B.A., Zucker-Franklin, D., and Coutavas, E.E. (1995). The cutaneous T cell lymphoma, mycosis fungoides, is a human T cell lymphotropic virus-associated disease. A study of 50 patients. *J Clin Invest* 95**,** 547-554.

Park, S., Lee, D.Y., Kim, W.S., and Ko, Y.H. (2010). Primary cutaneous Epstein-Barr virus-associated T-cell lymphoproliferative disorder-2 cases with unusual, prolonged clinical course. *Am J Dermatopathol* 32**,** 832-836.

Phan, A., Veldman, R., and Lechowicz, M.J. (2016). T-cell Lymphoma Epidemiology: the Known and Unknown. *Curr Hematol Malig Rep* 11**,** 492-503.

Poiesz, B., Dube, D., Dube, S., Love, J., Papsidero, L., Uner, A., and Hutchinson, R. (2000). HTLV-II-associated cutaneous T-cell lymphoma in a patient with HIV-1 infection. *N Engl J Med* 342**,** 930-936.

Pomerantz, R.G., Campbell, L.S., Jukic, D.M., and Geskin, L.J. (2010). Posttransplant cutaneous T-cell lymphoma: case reports and review of the association of calcineurin inhibitor use with posttransplant lymphoproliferative disease risk. *Arch Dermatol* 146**,** 513-516.

Ponti, R., Bergallo, M., Costa, C., Quaglino, P., Fierro, M.T., Comessatti, A., Stroppiana, E., Sidoti, F., Merlino, C., Novelli, M., Alotto, D., Cavallo, R., and Bernengo, M.G. (2008). Human herpesvirus 7 detection by quantitative real time polymerase chain reaction in primary cutaneous T-cell lymphomas and healthy subjects: lack of a pathogenic role. *Br J Dermatol* 159**,** 1131-1137.

Safai, B., Myskowski, P.L., Dupont, B., and Pollack, M.S. (1983). Association of HLA-DR5 with mycosis fungoides. *J Invest Dermatol* 80**,** 395-397.

Schmidt, A.N., Robbins, J.B., Greer, J.P., and Zic, J.A. (2006). Conjugal transformed mycosis fungoides: the unknown role of viral infection and environmental exposures in the development of cutaneous T-cell lymphoma. *J Am Acad Dermatol* 54**,** S202-205.

Shohat, M., Hodak, E., Hannig, H., Bodemer, W., David, M., and Shohat, B. (1999). Evidence for the cofactor role of human T-cell lymphotropic virus type 1 in mycosis fungoides and Sezary syndrome. *Br J Dermatol* 141**,** 44-49.

Shuda, M., Arora, R., Kwun, H.J., Feng, H., Sarid, R., Fernandez-Figueras, M.T., Tolstov, Y., Gjoerup, O., Mansukhani, M.M., Swerdlow, S.H., Chaudhary, P.M., Kirkwood, J.M., Nalesnik, M.A., Kant, J.A., Weiss, L.M., Moore, P.S., and Chang, Y. (2009). Human Merkel cell polyomavirus infection I. MCV T antigen expression in Merkel cell carcinoma, lymphoid tissues and lymphoid tumors. *Int J Cancer* 125**,** 1243-1249.

Sidoti, F., Fierro, M.T., Costa, C., Ponti, R., Bergallo, M., Comessatti, A., Fumagalli, M., Novelli, M., Merlino, C., Cavallo, R., and Bernengo, M.G. (2009). Prevalence and significance of human parvovirus variants in skin from primary cutaneous T cell lymphomas, inflammatory dermatoses and healthy subjects. *Arch Dermatol Res* 301**,** 647-652.

Tan, R.S., Butterworth, C.M., Mclaughlin, H., Malka, S., and Samman, P.D. (1974). Mycosis fungoides--a disease of antigen persistence. *Br J Dermatol* 91**,** 607-616.

Tokura, Y., Heald, P.W., Yan, S.L., and Edelson, R.L. (1992). Stimulation of cutaneous T-cell lymphoma cells with superantigenic staphylococcal toxins. *J Invest Dermatol* 98**,** 33-37.

Tokura, Y., Yagi, H., Ohshima, A., Kurokawa, S., Wakita, H., Yokote, R., Shirahama, S., Furukawa, F., and Takigawa, M. (1995). Cutaneous colonization with staphylococci influences the disease activity of Sezary syndrome: a potential role for bacterial superantigens. *Br J Dermatol* 133**,** 6-12.

Tournadre, A., D'incan, M., Dubost, J.J., Franck, F., Dechelotte, P., Souteyrand, P., and Soubrier, M. (2001). Cutaneous lymphoma associated with Epstein-Barr virus infection in 2 patients treated with methotrexate. *Mayo Clin Proc* 76**,** 845-848.

Tuyp, E., Burgoyne, A., Aitchison, T., and Mackie, R. (1987). A case-control study of possible causative factors in mycosis fungoides. *Arch Dermatol* 123**,** 196-200.

Weinstock, M.A., and Gardstein, B. (1999). Twenty-year trends in the reported incidence of mycosis fungoides and associated mortality. *Am J Public Health* 89**,** 1240-1244.

Wilkins, K., Turner, R., Dolev, J.C., Leboit, P.E., Berger, T.G., and Maurer, T.A. (2006). Cutaneous malignancy and human immunodeficiency virus disease. *J Am Acad Dermatol* 54**,** 189-206; quiz 207-110.

Zucker-Franklin, D., Hooper, W.C., and Evatt, B.L. (1992). Human lymphotropic retroviruses associated with mycosis fungoides: evidence that human T-cell lymphotropic virus type II (HTLV-II) as well as HTLV-I may play a role in the disease. *Blood* 80**,** 1537-1545.
